# Impact of chromium dinicocysteinate supplementation on inflammation, oxidative stress, and insulin resistance in type 2 diabetic subjects: an exploratory analysis of a randomized, double-blind, placebo-controlled study

**DOI:** 10.3402/fnr.v60.31762

**Published:** 2016-09-28

**Authors:** Zainulabedin M. Saiyed, James P. Lugo

**Affiliations:** InterHealth Nutraceuticals, Benicia, CA, USA

**Keywords:** chromium dinicocysteinate, diabetes, insulin resistance, vascular inflammation, oxidative stress

## Abstract

**Background:**

Chromium dinicocysteinate (CDNC) is a unique chromium complex consisting of chromium, niacin, and L-cysteine. Previous preclinical and clinical studies support the safety and efficacy of CDNC in modulating oxidative stress, vascular inflammation, and glycemia in type 2 diabetes.

**Objective:**

Herein, we report the results of several exploratory analyses conducted on type 2 diabetic subjects who previously participated in a 3-month randomized, double-blind, placebo-controlled trial and were treated with only metformin as standard diabetic care in addition to receiving the test supplementations.

**Design:**

Results from 43 metformin users, who were randomly assigned to receive either placebo (P, *n*=13), chromium picolinate (CP, 400 µg elemental Cr^3+^/day, *n*=12), or CDNC (400 µg elemental Cr^3+^/day, *n*=18), were analyzed for blood markers of vascular inflammation, insulin resistance, and oxidative stress at baseline and at 3 months of supplementation.

**Results:**

A statistically significant decrease in insulin resistance in the CDNC-supplemented cohort compared to placebo (*p*=0.01) was observed at 3 months. The CDNC group also demonstrated a significant reduction in insulin levels (*p*=0.03), protein carbonyl (*p*=0.02), and in TNF-α (*p*=0.03) compared to the placebo group. The CP group only showed a significant reduction in protein carbonyl levels (*p*=0.03) versus placebo.

**Conclusions:**

When controlling for diabetes medication, CDNC supplementation showed beneficial effects on blood markers of vascular inflammation, insulin resistance, and oxidative stress compared to placebo. The findings suggest that CDNC supplementation has potential as an adjunct therapy for individuals with type 2 diabetes.

The Diabetes Control and Complication Trial showed that tight blood glucose control considerably slows the onset and progression of diabetes-associated complications (1, 2). However, many diabetic patients struggle to manage their blood glucose levels with existing treatment regimens. Experimental evidence suggests that, for diabetic patients, a subclinical chromium deficiency is linked to elevated blood glucose, insulin, and lipid levels that may interfere with the management of diabetes (3–5).

Preclinical and clinical studies suggest that trivalent chromium plays an important role in improving glucose and lipid metabolism (6–10). Estimates indicate that about 10 million Americans use chromium supplements daily for the prevention or treatment of diabetes (11).

Chromium dinicocysteinate (CDNC), a complex of chromium, niacin, and L-cysteine, has been shown to exert beneficial effects on decreasing levels of fasting glucose, insulin, glycated hemoglobin, and vascular inflammation markers in zucker diabetic fatty (ZDF) rats (12). Jain et al. (13) undertook a randomized, double-blind, placebo-controlled clinical study to evaluate the safety and efficacy of CDNC in lowering oxidative stress, vascular inflammation, and insulin resistance in type 2 diabetic subjects. The results of that study showed a significant decrease in insulin resistance, protein oxidation, and TNF-α levels in CDNC-supplemented subjects at 3 months compared to baseline values. Here, we report the results of several exploratory analyses from this prior study that assessed the impact of supplementation with CDNC in type 2 diabetic subjects treated with metformin only.

## Methods

### Study subjects

Type 2 diabetic subjects, aged 30–55 years, were recruited into this study. Subjects with a history of cardiovascular disease, sickle cell disease, treatment with insulin, or metabolic disorders, including uncontrolled hypertension, hypothyroidism, or hyperthyroidism, were excluded. Detailed inclusion–exclusion criteria have been described previously (13). The study protocol was approved by the Louisiana State University Health Sciences Center’s Institutional Review Board.

### Study design

Exploratory analyses were performed using data from the Jain study (13). Subjects were stratified according to the medical treatment received for managing diabetes at the start of the study. The majority of subjects were treated with metformin (see Table 1).

**Table 1:** Number of subjects stratified based on medication usage

Medication regimen	Placebo (*n*=25) (% of subjects)	CDNC (*n*=24) (% of subjects)	CP (*n*=25) (% of subjects)
Metformin	13 (52)	18 (75)	12 (48)
Metformin+Januvia	2 (8)	None	None
Metformin+Glipizide	6 (24)	2 (8.3)	5 (20)
Metformin+Glyburide	2 (8)	4 (16.7)	4 (16)
Metformin+Januvia+Glyburide	None	None	1 (4)
Avandia	None	None	1 (4)
No medication	2 (8)	None	2 (8)

The study consisted of a 1-month placebo run-in period followed by random assignment to one of three groups: placebo (P), chromium picolinate (CP, 400 µg Cr^3+^/day), or CDNC (400 µg Cr^3+^/day) for 3 months. Subjects continued to receive standard medical care for diabetes during the study.

### Measurement of biomarkers in plasma

TNF-α and insulin levels were determined using ELISA kits from Thermo Fisher Scientific Inc. (Rockford, IL). Protein oxidation was assessed by determining protein carbonyl levels using an ELISA kit from ENZO^®^ Life Sciences International Inc. (Plymouth Meeting, PA). Glucose levels were estimated using an ACCU-CHEK^®^ Advantage glucometer (Boehringer-Mannheim, Indianapolis, IN). Insulin resistance was calculated from glucose and insulin levels using the HOMA insulin resistance (HOMA-IR) method as described previously by Yaturu et al. (14).

### Statistical analysis

The Kruskal–Wallis test was used to compare the mean changes among the three groups. The Wilcoxon rank-sum test was used to determine pairwise statistical difference on mean changes among the groups. For within-group comparisons, the Wilcoxon-signed rank test was used to determine any significant changes between the baseline and 3 months. Non-parametric statistical methods were used due to the observed non-normality of the differences within each group. SAS^®^ Version 9.3 software (Cary, NC) was used for the statistical analysis. *p* values ≤0.05 were considered statistically significant. Statistical analyses were done by an independent statistician at the Louisiana State University Health Sciences Center.

## Results

### Baseline demographic characteristics

Overall, the subject profiles with respect to age, weight, body mass index (BMI), glucose, insulin, TNF-α, protein carbonyl, and hemoglobin (Hb) A1c levels were comparable among the three study groups (Table 2).

**Table 2:** Demographic and baseline characteristics of metformin using subjects

	Placebo (*n*=13)	CDNC (*n*=18)	CP (*n*=12)	*p* value
Age (years)	46.9±3.2	49.3±2.1	50.6±3.3	0.461
Weight (kg)	99.9±8.5	101.1±8.0	103.9±7.2	0.638
Fasting glucose (mg/dL)	138.8±21.2	130.2±11.7	110.8±6.9	0.497
HbA1c (%)	7.6±0.6	7.5±0.4	6.9±0.3	0.595
Insulin (µU/mL)	25.5±4.6	32.5±5.2	25.8±4.2	0.684
TNF-α (pg/mL)	6.5±1.1	5.9±0.3	5.9±0.8	0.869
Protein carbonyl (OD/plasma)	0.38±0.03	0.40±0.02	0.39±0.04	0.819
Insulin resistance (HOMA)	7.9±1.6	9.9±1.7	7.0±1.1	0.674

Values are expressed as mean±SEM. *p* values were obtained by comparing the mean values among the three groups using the Kruskal–Wallis test. No significant differences were observed among the groups.

### Reduction in plasma insulin and glucose levels

Table 3 summarizes the changes in insulin levels from baseline to 3 months for subjects supplemented with either CDNC, CP, or placebo. The CDNC group experienced a statistically significant decrease in insulin levels compared to the placebo group (−8.80 vs. 3.73 µU/mL, *p*=0.03). This decrease for the CDNC group, coupled with a net increase in insulin levels for placebo, resulted in a significant net change of 12.53 µU/mL at the study’s conclusion. By contrast, no significant difference in insulin levels was seen in the placebo or the CP group.

**Table 3:** Mean changes in fasting glucose and insulin levels plus insulin resistance from baseline to 3 months among metformin-using subjects

			*p* value			
			
Variable	Study groups (*n*)	Average change	Overall^a^	Within group^b^	vs. placebo^c^	vs. CP^c^
Glucose (mg/dL)	Placebo (13)	−6.62±12.65	0.20	0.66	–	–
	CDNC (18)	−10.50±3.81		0.02*	0.10	0.20
	CP (12)	6.75±13.81		0.92	0.66	–
Insulin (µU/mL)	Placebo (13)	3.73±3.21	0.08	0.31	–	–
	CDNC (18)	−8.80±3.49		0.04*	0.03*	0.20
	CP (12)	−1.93±2.57		0.73	0.30	–
Insulin resistance (HOMA)	Placebo (13)	1.35±1.22	0.02*	0.30	–	–
	CDNC (18)	−3.52±1.20		0.01*	0.01*	0.0515
	CP (12)	0.65±1.53		0.73	0.38	–

Values are expressed as mean±SEM.

a Overall *p* value was obtained by comparing the mean changes among the three groups using the Kruskal–Wallis test.

b The Wilcoxon signed-rank test was used to determine changes between the baseline and 3 months.

c The Wilcoxon rank-sum test was used to determine pairwise differences on mean changes among the groups.

* Significant at 5% level (*p*≤0.05).

A significant reduction in fasting blood glucose level was observed in the CDNC group (−10.50 mg/dL, *p*=0.02) versus the baseline value (Table 3). By contrast, no significant change was seen in subjects supplemented with placebo or CP. After 3 months of CDNC supplementation, the average fasting glucose values declined to a pre-diabetes range (130.2 vs. 119.7 mg/dL, *p*=0.02, Table 3). No such decrease was observed for either the CP or the placebo cohort.

### Reduction in insulin resistance

As shown in Table 3, a statistically significant decrease in insulin resistance was observed for subjects consuming CDNC compared to placebo (−3.52 vs. 1.35 HOMA, *p*=0.01). A strong trend toward statistical significance was seen in the CDNC-supplemented cohort versus the CP group (−3.52 vs. 0.65 HOMA, *p*=0.0515). No significant change in insulin resistance was seen for either the placebo or the CP cohort.

### Modulation of plasma TNF-α and protein carbonyl levels

Changes in the levels of TNF-α and protein carbonyl for metformin-only users are summarized in Table 4. The results show that CDNC supplementation for 3 months yielded a statistically significant reduction in TNF-α levels compared to placebo (−1.04 vs. 0.77 pg/mL, *p*=0.03). No significant change in TNF-α levels was noted after placebo or CP supplementation. The CDNC-supplemented group also exhibited a statistically significant decrease in protein carbonyl levels compared to the placebo group (−0.03 vs. 0.03 OD, *p*=0.02). Similarly, CP supplementation resulted in a significant reduction in protein carbonyl levels compared to placebo (−0.05 vs. 0.03 OD, *p=*0.03) (Table 4). Pairwise comparison showed no significant difference between CDNC and CP groups for either TNF-α or protein carbonyl levels.

**Table 4:** Mean changes in blood TNF-α and protein carbonyl levels from baseline to 3 months among metformin-using subjects

			*p value*			
			
Variable	Study groups (*n*)	Average change	Overall^a^	Within group^b^	vs. placebo^c^	vs. CP^c^
TNF-α (pg/mL)	Placebo (13)	0.77±1.11	0.15	0.99	–	–
	CDNC (18)	−1.04±0.54		0.005**	0.03*	0.87
	CP (12)	−1.19±0.86		0.27	0.34	–
Protein carbonyl (OD/plasma)	Placebo (13)	0.03±0.02	0.033*	0.11	–	–
	CDNC (18)	−0.03±0.02		0.08	0.02*	0.77
	CP (12)	−0.05±0.03		0.07	0.03*	–

Values are expressed as mean±SEM.

a Overall *p* value was obtained by comparing the mean changes among the three groups using the Kruskal–Wallis test.

b The Wilcoxon signed-rank test was used to determine changes between the baseline and 3 months.

c The Wilcoxon rank sum test was used to determine pairwise differences on mean changes among the groups.

* Significant at 5% level (*p*≤0.05).

** Significant at 1% level (*p*≤0.01).

## Discussion

The objective of the current analyses was to examine the effect of CDNC supplementation on oxidative stress, insulin resistance, and on markers of vascular inflammation in type 2 diabetic subjects who were treated with metformin as their standard of care. The rationale behind this approach centered on the idea that, as diabetes progresses, subjects require multiple medications to maintain blood sugar control due to a dwindling responsiveness to intervention (15). We therefore stratified the subjects from the Jain et al. (13) study to include only those individuals on metformin in order to control for this confounding variable.

In these exploratory analyses, the data demonstrated that supplementation with CDNC markedly reduced insulin (27.1%) and insulin resistance (35.4%) compared to the baseline levels. The CDNC-treated group achieved statistical significance versus placebo and showed a strong statistical trend versus CP for insulin resistance. One contributing factor for the change in insulin resistance for the CDNC group was a significant decrease in blood glucose levels (8.1%) versus baseline value. This outcome contrasts sharply with that of the CP cohort, which presented at study conclusion with a net increase in blood glucose levels and insulin resistance of 6.1 and 9.3%, respectively.

Studies report that blood levels of TNF-α and oxidative stress are higher in diabetic subjects and are indicative of an ongoing inflammatory response that ultimately results in development of cardiovascular disease (16–18). The current analysis showed that CDNC supplementation significantly reduced the blood levels of inflammatory and oxidative stress markers versus placebo. By contrast, supplementation with CP led to a significant reduction in oxidative stress only compared to placebo.

The impact of CDNC supplementation on blood glycemic biomarkers, inflammation, and on oxidative stress levels corroborates with preclinical findings that CDNC supplementation for 8 weeks significantly moderated glucose, glycated hemoglobin, inflammatory markers, and lipid peroxidation levels in ZDF rats (12). The results from this previous animal study provides insights as to the possible mechanism by which CDNC mediates its beneficial effects. It indicates that CDNC works in part by elevating blood adiponectin and vitamin C levels, plus suppressing NFκB, Akt, and Glut-2 levels, and activating insulin receptor substrate (IRS-1) in the liver (12). The modulation of these markers is suggestive of an improvement in insulin signaling. Additional studies that elucidate the precise mechanism through which CDNC improves insulin sensitivity are required.

A key limitation of the current analyses is that the original study (13) was not sufficiently powered for stratification based on medication usage. Nevertheless, the results of these exploratory analyses underscore a definite trend indicating that CDNC supplementation may be beneficial in managing insulin resistance. These encouraging results suggest that future CDNC investigations in larger, powered studies are warranted.

## Conclusions

In conclusion, the exploratory analyses indicate that, when controlling for diabetes medication, supplementation with CDNC appears to improve insulin sensitivity by reducing blood levels of insulin and glucose as well as the oxidative stress and inflammation associated with diabetes.
